# A seat at the table: integrating gender and structural reform to ensure the right to food

**DOI:** 10.1017/S1368980026102109

**Published:** 2026-02-11

**Authors:** Marichu Butaslac Montecillo, Madelaine Suarez Dumandan, Loreta Sol Litanon Dinlayan

**Affiliations:** Bukidnon State University, Malaybalay City, Bukidnon, Philippines

**Keywords:** Food, Income, Gender equality, Public policy, Food security

Food security exists when people have access to enough safe and nutritious food for normal growth and development and an active and healthy life^(1)^. The core factors that affect food security are use or utilisation, access, availability and stability^(1–3)^.

The study of Rodrigues *et al.* ^(4)^ on food security and employment income provides essential, nationally representative evidence from the 2018 Brazilian Family Budget Survey (POF) on the critical intersection of gender, employment income and household food insecurity (FI). The study published in 2025 showed that being food insecure can be determined by employment income and if the family is headed by a male or female. In a country where the struggle to guarantee food security remains persistent, this study is a powerful contribution to the ongoing public health and policy debate.

According to the World Food Program^(5)^, food security exists when people have access to enough safe and nutritious food for normal growth and development and to lead an active and healthy life. The four core factors influencing food security are availability, accessibility, utilisation and stability^(1,3)^. Availability depends on production, stock levels and trade, while accessibility relates to household income, expenditure and market conditions. Utilisation refers to people’s ability to safely prepare and absorb nutrients from food, which requires clean water and fuel. Stability, a crosscutting dimension, is tied to vulnerabilities such as inflation, extreme weather and political instability. The State of Food Security and Nutrition in the World^(2)^ underscores how rising inflation has reduced purchasing power, limiting access to healthy diets, especially for low-income households.

The study by Rodrigues *et al.* ^(4)^ confirms that household income remains a central determinant of food accessibility, a finding that aligns closely with the work of De Marco *et al.* ^(3)^. Beyond income alone, the gender of the household head significantly influences vulnerability. This is consistent with Drammeh *et al.* ^(6)^, who observe that FI is inextricably linked to poverty, education, household size, employment status and food price fluctuations. Specific evidence from Nigeria and South Africa further illustrates this disparity, noting that female-headed households often face higher risks of FI than those headed by men^(7)^. However, research consistently demonstrates that empowering women through enhanced economic status, education and decision-making power acts as a catalyst for agricultural productivity and improved household nutrition.

This vulnerability is not uniform, as the structure of female-headed households is increasingly diverse and does not always stem from traditional crises like widowhood or divorce. While Horrell and Krishnan^(8)^ acknowledge that many such households result from marital dissolution, they also highlight a rising trend of ‘de facto’ or ‘choice-based’ headship. In these instances, women assume the role because they are the primary breadwinners or hold more stable employment than their partners. Across various regions, this shift reflects a fundamental change in gendered power dynamics, where female headship emerges from economic mobility and deliberate choice rather than domestic instability. Such structural transformations suggest a broader societal move towards gender symmetry^(9)^.

Rodrigues *et al.* ^(4)^ strengthens this discourse by utilising a robust methodological framework. Notably, it is among the first to use POF data to link employment income and FI specifically through the gender of the reference person, offering a more granular analysis than total family income alone. The reliability of these findings is bolstered by the use of the Brazilian Food Insecurity Scale (EBIA), a validated, experience-based measure. Furthermore, by adopting an explicit intersectional gender perspective, the study moves beyond binary analysis to address the power relations structuring inequality. Finally, the use of a multivariate logistic regression model ensures that the results are adjusted for critical confounders, including geographic region, race/skin colour and participation in the Bolsa Família Program.

However, the study also highlights three key methodological gaps that offer fertile ground for improvement in future research. One is the exclusion of the formal/informal status of the job. The study was limited in that it did not consider whether the employment income of the reference person stemmed from a formal or an informal job. This omission is significant, as women are more likely to be engaged in informal, unstable employment, which is a major contributor to precarious income and household FI. Future analyses of the POF data should attempt to stratify the employment income variable by formality of the job to more precisely measure the resulting gender inequality in labour income. Studies in other contexts, such as Peru, have shown that female-headed households have a higher proportion of informally employed members, increasing their FI vulnerability.

Second, the study’s reliance on the pre-COVID-19 pandemic 2018 Family Budget Survey data significantly limits its ability to accurately represent current socio-economic realities, particularly concerning income and gender inequality. The global health crisis has dramatically reshaped employment landscapes, income stability and household structures, making the 5-year-old data potentially obsolete for effective policy-making. To ensure that the study’s insights are a credible basis for policy formulation, it is critically important to utilise more recent data that accurately captures the complex and evolving associations between income from work, the gender of the household’s reference person and the pervasive challenges of family and FI. The relevance of incorporating the recent changes – precipitated by a major contributory factor like a health crisis – cannot be overstated, as outdated statistics risk leading to policies that are misaligned with the actual needs and vulnerabilities of the contemporary population.

The third issue is the ambiguity surrounding the definition of the ‘Reference Person’. The authors acknowledge that the definition of the household reference person in IBGE surveys is often ambiguous due to changing family structures, making it difficult to establish the exact reasons for headship (e.g. contributing the largest income share *v*. taking care of the house). This limitation constrains the ability to definitively link headship to specific domestic responsibilities. A necessary improvement would be to complement such large-scale quantitative data with qualitative studies that explore the negotiation and declaration of household headship in different family structures.

In conclusion, while Rodrigues and colleagues successfully demonstrate the nexus between employment income and FI, future efforts should holistically integrate the multidimensional determinants of food security. Addressing gender inequities alongside structural drivers such as inflation, education and household dynamics is essential for building resilient, food-secure communities.
